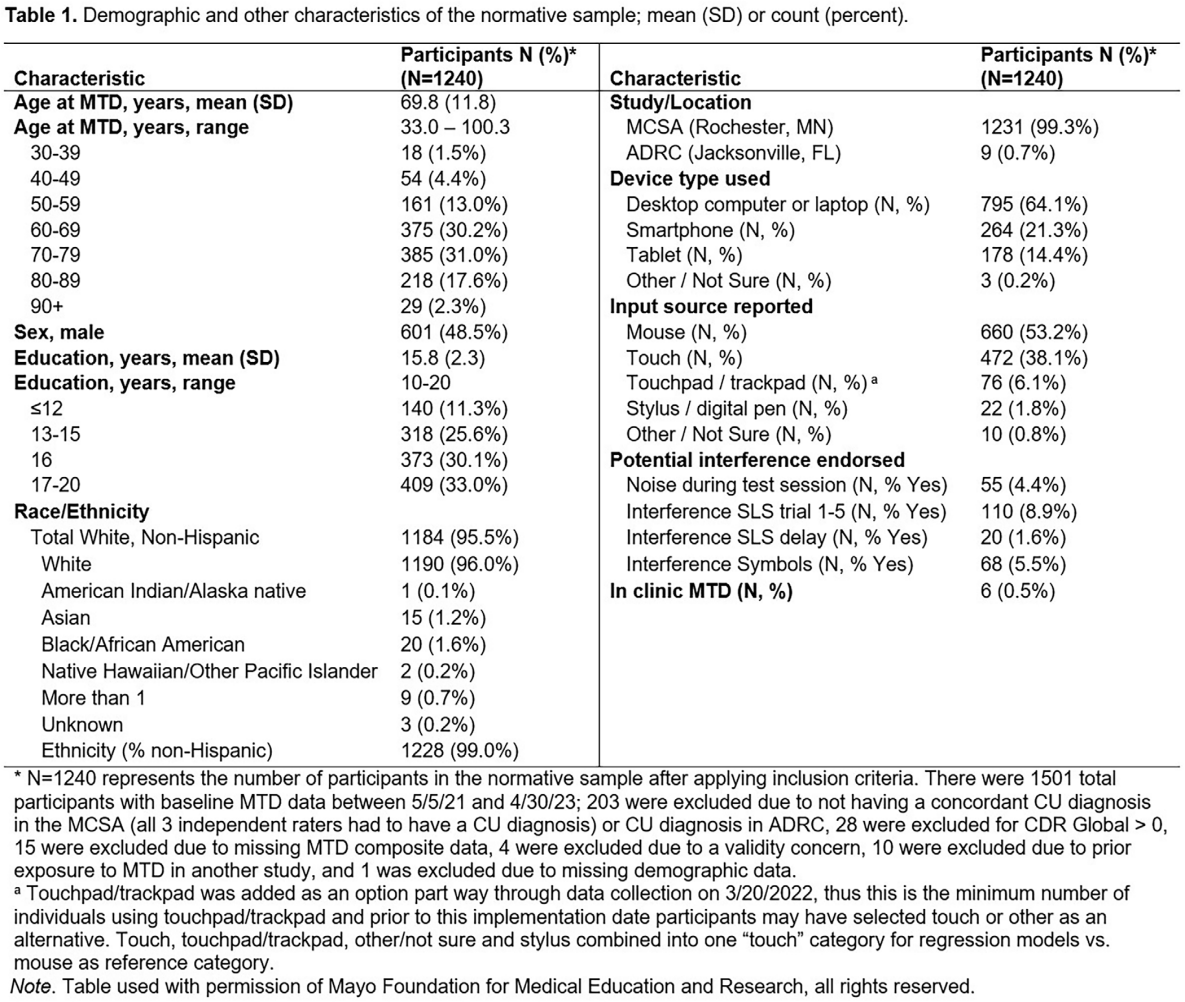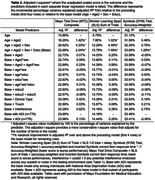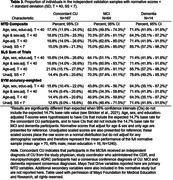# Mayo Normative Studies: regression‐based normative data for remote self‐administration of the Stricker Learning Span, Symbols Test and Mayo Test Drive Screening Battery Composite for ages 33‐100 and validation in MCI and dementia

**DOI:** 10.1002/alz.094197

**Published:** 2025-01-09

**Authors:** Nikki H. Stricker, Ryan D. Frank, Elizabeth A. Boots, Winnie Z. Fan, Teresa J. Christianson, Walter K. Kremers, John L. Stricker, Mary M. Machulda, John A. Lucas, Jason J. Hassenstab, Jonathan Graff‐Radford, Prashanthi Vemuri, Clifford R. Jack, David S. Knopman, Ronald C. Petersen

**Affiliations:** ^1^ Mayo Clinic, Rochester, MN USA; ^2^ Mayo Clinic, Jacksonville, FL USA; ^3^ Washington University St. Louis, St. Louis, MO USA

## Abstract

**Background:**

Few normative data for computerized measures administered in unsupervised remote environments are available. We aimed to determine what variables to include in normative models for remote self‐administered assessments, develop normative data for measures administered through Mayo Test Drive (MTD, a multi‐device remote cognitive assessment platform) and evaluate application of norms.

**Method:**

1240 adults ages 33‐100 (96% White) from the Mayo Clinic Study of Aging and Mayo Alzheimer’s Disease Research Center met normative sample inclusion criteria that included a concordant Cognitively Unimpaired (CU) diagnosis (3 independent raters all diagnosed CU) and CDR = 0 (see Table 1 for sample characteristics). Raw test scores were converted to normalized scaled scores. We derived regression‐based normative data that adjusted for age, age2, sex and education (base model); alternative models are also provided (age+age2+sex; age+ age2). We evaluated whether additional model terms beyond the base model were needed using an a priori cut‐off of 1% variance improvement in adjusted R2 over and above the base model. We examined rates of low test performance defined as performance below ‐1 SD in independent validation samples (n = 167 concordant CU, n = 64 mild cognitive impairment (MCI), n = 14 dementia). Rates are considered significantly different than expected when 95% confidence intervals (CIs) do not include the expected 14.7% base rate frequency.

**Result:**

No additional potential model terms met our a priori cut‐off for inclusion beyond the base model (see Table 2), including other quadratic and cubic terms or interactions, device type, response input type, potential session interference, and national area deprivation index (ADI available in subset; N = 776). Application of norms showed expected rates of low test performance among CU participants (95% CI includes expected base rate) and significantly greater than expected rates of low test performance for all variables in MCI (e.g., 62.5% for composite) and dementia (e.g., 71.4% for composite; see Table 3).

**Conclusion:**

Typical normative models appear appropriate for remote self‐administered measures and are sensitive to cognitive impairment. Device type and response input did not explain enough variance to warrant inclusion in normative models but are important for individual level interpretation. Future work will improve representation of individuals from under‐represented groups.